# EGFR Targeting in Hormone-Refractory Prostate Cancer: Current Appraisal and Prospects for Treatment

**DOI:** 10.3390/ph3072238

**Published:** 2010-07-19

**Authors:** Olivier Guérin, Jean Louis Fischel, Jean-Marc Ferrero, Alexandre Bozec, Gerard Milano

**Affiliations:** 1 Oncopharmacology Unit, Centre Antoine Lacassagne, 33 Ave Valombrose, 06100 Nice, France; 2 Department of Gerontology, Centre Hospitalier et Universitaire de Nice, Hôpital de Cimiez, 4 Ave Reine Victoria, 06000 Nice, France; 3 University of Nice Sophia-Antipolis, Nice, France

**Keywords:** chemotherapy, targeted therapy, EGFR, kinase inhibitors, prostate cancer, hormone-resistant prostate cancer, geriatric oncology

## Abstract

The incidence of prostate cancer increases with age and because of its high prevalence this disease has become a major public health concern. Despite advances in our understanding of the biological mechanisms responsible for the development of this cancer, the transition to the hormone refractory stage (HRPC) and metastatic progression pose real problems of clinical management. Currently, docetaxel chemotherapy has been shown to have a slight but significant impact on survival, though the gain in median survival is still less than three months. Research is therefore continuing to improve treatment outcomes. The progression of prostate cancer is accompanied by the overexpression of EGFR (epidermal growth factor receptor) in a very large majority of cases, suggesting that this may play a mechanistic role. Unfortunately, although preclinical findings seem to be promising for therapies targeting the EGFR in HRPC, current clinical results are disappointing. These results should however encourage us to look for different ways of using anti-EGFR agents or combining them with other targeted therapies.

## 1. Introduction

Prostate cancer, which is the first male cancer in France, has benefited from advances in surgery, chemotherapy, radiotherapy and hormone therapy during the last ten years. In 2008 in the United States, prostate cancer accounted for 25% of new cases of cancer [1], and 60,000 new cases in France. As this cancer often occurs in senior adult men, any therapeutic strategy must take quality of life into account. However, when the cancer becomes hormone-refractory (HRPC), a new stage in disease progression has been crossed, and chemotherapies must be reassessed. Although past therapy combining mitoxantrone and prednisone did not improve patient survival, it does relieve pain and improve quality of life [2]. Currently, the reference treatment for hormone-resistant prostate cancer combines docetaxel and prednisone [3]. However the median increase in survival acheived by this treatment is only 2.9 months (median survival of 19.2 months *vs.* 16.3 months with mitoxantrone, p = 0.004). Future strategies aimed at improving management of hormone-refractory advanced prostate cancer should examine the benefits of combining other agents with docetaxel. The current responses to second line-treatments are disappointing and considerable progress remains to be made. This literature review summarizes our understanding of the merits of treatments targeting EGFR in this setting, as current data about these agents require our attention.

## 2. EGFR: A Particularly Interesting Target in Oncology

Epidermal growth factor receptor (EGFR or HER-1) was the first known member of the HER receptor family. In the physiological state, EGFR is expressed by many epithelia (skin, cervix, bile ducts, hepatocytes, sebaceous glands, bronchi, bladder, breast myoepithelial cells). Likewise, EGFR is overexpressed in many cancers (non-small cell lung cancer, head and neck cancer, ovarian, colon, bladder, kidney and prostate cancers) [4].

EGFR is a receptor with a tyrosine kinase activity. It plays a key role in the signal transduction processes, controlling major cell functions, such as survival and proliferation. Activation of EGFR signaling requires the binding on this receptor of growth factors such as EGF, TGFα, amphiregulin, heparin-binding EGF, and betacellulin (although EGF and TGFα are the preferred ligands) [5], leading to its dimerization or heterodimerization with other receptors of HER family (HER-2 in particular, but also HER 3 and HER 4). The autophosphorylation and transphosphorylation of receptors via their tyrosine kinase domains then leads to the recruitment of intracellular effectors and to the activation of the proliferation and survival pathways [6].

Targeted therapies in oncology currently include two main categories of molecules: monoclonal antibodies (Acm) and tyrosine kinase inhibitors (TKI) [7]. The best known agents targeting EGFR, with the most advanced clinical development includecetuximab (Erbitux^®^) [8,9] for Acm andgefitinib (Iressa^®^) [10,11] or erlotinib (Tarceva^®^) [12,13] for TKI. Many other molecules are also under clinical or preclinical development and, in particular for TKI, these are now also multitargeted as EGFR is not the only receptor involved in their action mechanisms. Acm and TKI clearly differ by their action mechanism on their target. Cetuximab is a competitive antagonist of EGF on its receptor. Independently of the phosphorylation of the receptor, the cetuximab-EGFR complex is then internalized [14,15]. On the contrary, TKI act on the cytosolic portion of EGFR, in competition with ATP at the level of its binding domain, thereby preventing the autophosphorylation of the receptor. Depending on the nature of the TKI, the inhibition of EGFR may be reversible as is the case with gefitinib and erlotinib, or irreversible as for example with PD-183805 [16,17,18]. Unlike Acm, TKI are not strictly specific for their supposed target (EGFR for example). As TKI are competitive antagonists of ATP at the level of its tyrosine kinasebinding sites [17], there may be a variable cross-reactivity of TKI with other members of the HER receptor family, such as HER-2 [19].

The effects of EGFR targeting testify to the physiological role of this receptor in the signal transduction pathways involved in cell division, apoptosis and neoangiogenesis. At the level of cell proliferation first of all, a slowing of cell division is observed with blocking of cells in the G1 phase, involving molecular changes at the main points of control of the cell cycle [20,21]. In addition, a change in the equilibrium between intracellular Bax and Bcl-2 levels has been reported, underlining the pro-apoptotic effect of EGFR targeting [22]. The anti-angiogenic effect of EGFR targeting was demonstrated for Acm and TKI, in particular by inhibition of tumor secretion of pro-angiogenic factors such as VEGF and factor VIII [23,24].

Studies performed *in vivo* on tumor xenografts show that cetuximab has a much greater efficacy than that observed on cell lines *in vitro* [25]. A significant part of the antitumor activity of Acm may be due to immunological processes such as antibody-dependant cell cytotoxicity (ADCC) [26,27]. These differences between the *in vitro* and *in vivo* activity noted with cetuximab are not as marked with TKI. Hence gefitinib and other more recently developed TKIs such as sunitinib (Sutent^®^), cause an inhibition of cell proliferation both *in vitro* and *in vivo*, on many cell lines [28,29].

Different studies have investigated the effects of combining anti-EGFR with conventional cytotoxic agents and radiotherapy. Additive or supra-additive cytotoxic effects are usually obtained with both Acm or TKI. At the experimental level, there seems to be no difference between Acm and TKI concerning the possibility of obtaining synergiccytotoxic effects, when they are combined with conventional chemotherapy agents or radiation. This synergy may be mainly assigned to the known effects of EGFR targeting on cell proliferation, apoptosis, angiogenesis and DNA repair [28,30,31,32].

At a clinical level, gefitinib and erlotinib for TKI, are used in the treatment of non-small cell lung cancer, where they were found to be particularly effective in patients with certain EGFR mutations [33,34]. Concerning Acm, cetuximab has currently been shown to be beneficial in the treatment of colorectal cancers, in combination with irinotecan [35]. Its efficacy has also been demonstrated in the treatment of squamous cell carcinomas of the upper respiratory and digestive tracts [36,37].

## 3. EGFR in Prostate Cancer and Its Outcome

This approach seemed particularly interesting for the treatment of prostate cancer. Indeed, numerous molecular mechanisms are linked to the transition to hormone-refractory prostate cancer [38]. However, the increased expression of HER signaling family proteins seems to be one important factor [39].

In a recent study, Schlomm *et al.* [40] analyzed DNA and protein levels in tissue samples from 2,497 prostate tumors. Detectable EGFR expression was found in 18% of cancers and was associated with high grade, advanced stages, and high risk for prostate-specific antigen recurrence by univariate analysis (p < 0.0001, each). The potential utility of anti-EGFR treatments could be analyzed in EGFR-expressing prostate cancer.

In addition, after total prostatectomy, the expression of EGFR is correlated with the risk of recurrence and progression to hormone resistance. By multivariate analysis, EGFR is an independent prognostic factor of progression-free survival. One hundred % of metastases of hormone-refractory prostate cancers express EGFR, suggesting that this receptor is a major transduction pathway for tumor growth [41].

In another study [42], EGFR amplification and mutation were analyzed in 10 patients with either hormone-sensitive or hormone-refractory prostate cancer. No significant correlation was found between mutation status and the hormone sensitive or refractory status. However, the time to convert to HRPC was significantly shorter in patients with a mutation in the EGFR gene (p = 0.017). In this study, EGFR mutation did not appear to play a significant role in the hormone refractory pathway but was associated with prognosis. The small number of subjects in this study must also be taken into account.

## 4. Main Results of Preclinical Studies Targeting EGFR in HRPC

Recent studies suggest that castration-induced prostate involution may be caused by primary effects in the prostate vasculature. Hammarsten *et al.* [43], recently investigated if antivascular treatments may mimic the effects of castration. The experimental design used androgen-independent AT-1 prostate cancer cells. These cells were grown inside the ventral prostate of adult rats. Tumor-bearing animals were treated with a VEGFR 2 and EGFR inhibitor: ZD6474 (Astra Zeneca), and authors compared short-term effects of this treatment (after three days) with those induced by castration. The results showed that castration caused by decreased vascular density in the normal tissue surrounding the tumor increased tumor hypoxia and apoptosis, and moderately decreased tumor growth. ZD6474 treatment resulted in decreased tumor vascular density accompanied by increased tumor hypoxia, apoptosis, and decreased tumor growth, suggesting that castration and antiangiogenic therapy work through a similar mechanism. Interestingly, combined treatment by castration + ZD 6474 was more effective than castration and ZD 6474 alone in inducing tumor hypoxia, apoptosis, necrosis, and decreasing tumor vascular density. The authors concluded that this combined treatment could be a particularly effective way to treat prostate tumors.

Our team tested in experimental animals [44] the combination of an anti-EGFR tyrosine kinase inhibitor (SU11248) having anantiangiogenic action, cetuximab (anti-EGFR) and docetaxel. Each drug, administered as a single-agent, has comparable and moderate effects on tumor growth with approximately 50% inhibition at the end of the 3-week dosing schedule. Computed combination ratio (CR) values for tumor growth indicated supra-additive effects for the sunitinib-docetaxel and sunitinib-cetuximab combinations, and suggested additive effects only for the sunitinib-cetuximab-docetaxel combination. The effects on tumor growth were accompanied by a parallel reduction in tumor cell proliferation (Ki 67) and tumorvascularization (Von Willebrand factor). The sunitinib-docetaxel and sunitinib-docetaxel-cetuximab combinations had significantly higher pro-apoptotic effects (caspase-3 cleavage) than the other conditions. We therefore concluded that the supra-additive anti-tumor effects observed with the sunitinib-docetaxel combination might support innovative strategies in the management of advanced prostate cancer, using simultaneous treatments targeting EGFR.

Concerning these combined approaches, recent results on cell lines (DU145) and *in vivo* murine models showed the benefit of cytotoxicdrug associated to pro-apoptotic radioimmunotherapy (combining lutetium 177 and an anti-prostate antibody hu3S193). In this article [45], the maximum tolerated dose of radioimmunotherapy was determined. One very interesting result was that the combination of this treatment with a TKI (AG1478) at subtherapeutic doses gave a significant improvement in efficacy on *in vivo* models as after combination with docetaxel.

## 5. Results of the Main Clinical Studies of Therapies Targeting EGFR in HRPC

### 5.1. Single Agent Therapy Trials

Gravis *et al.* [46] performed a Phase II study on 30 patients including 29 HRPC. Twenty three patients had already received a first line of chemotherapy. They had a median PSA of 102 ng/mL (range 3–1,213 ng/mL). The patients received 150 mg/day of erlotinib for the first four weeks and then 200 mg/day if toxicity was acceptable. The primary endpoint was a reduction or stabilization of PSA with no clinical progression. Toxicity was moderate, but only 14% of patients had at least a stabilization of PSA. On the other hand, a clinical benefit was obtained in 40% of patients (defined by an improvement in the Karnofsky index or pain), which was an interesting result at this stage of the disease, though this was not the primary endpoint.

As mentioned above, Gefitinib is another molecule targeting EGFR. In a phase II study which included about 100 HRPC patients, no response and a very low anti-cancer activity was observed [47]. This resistance to gefitinib was perhaps due to an overactivity of the PI3K/Akt pathway in prostate cancer [48], and trials with combination strategies are therefore a priori more interesting.

A recent study [49] shown EGFR tyrosine kinaseindependant mechanisms on survival of prostate cancer cell through stabilization of SGLT1, a sodium cotransporter. In others words, the function of kinase-independant EGFR is to prevent autophagic cell death by maintaining intracellular glucose level through interaction and stabilization of SGLT1. Such mechanism could also explain HRPC resistance to TKI.

### 5.2. Trials of Treatment Combinations

Only few clinical trials have currently been performed using combinations with anti-EGFR agents. As we have seen Docetaxel is the standard first-line treatment for HRPC. Combination with targeted anti-EGFR therapies may improve efficacy and reduce toxicity and make it possible to reduce the doses of the antimitotic agent.

Erlotinib is an orally active and reversible inhibitor of EGFR tyrosine kinase that blocks the cell cycle in the G1 phase. Chiorean *et al.* [50] performed a study to define the maximum tolerated weekly dose of docetaxel combined with daily erlotinib. Twenty five patients were enrolled. Responses were seen in prostate cancer (and in non-small cell lung cancer and hepatocellular carcinoma), and the recommended doses for phase II were a weekly dose of 30 mg/m² of docetaxel and a daily dose of 150 mg of erlotinib.

Gross *et al.* used Erlonitib in HRPC [51]. Erlonitib is a specific inhibitor of the tyrosine kinase activity of EGFR. This was a multi-institutional phase II study in patients with HRPC aged > 65years. Twenty two patients were enrolled (median age 73.5 years). There was no objective response when there were measurable radiological targets (eight patients), but five patients had a >50% reduction in PSA. There were two febrile neutropenias and the most frequent toxicities were fatigue, anorexia and diarrhea. The anti-cancer disease activity was generally comparable to docetaxel when used as monotherapy. Hematologic and non-hematologic toxicity may be increased over than docetaxelmonotherapy. This combination therefore did not appear to have any benefit during this phase II study in an elderly population.

New molecules act on several targets, including EGFR. This is the case for Vandetanib, a once-daily oral anticancer drug that inhibits VEGF and REGF. Horti *et al*. [52] conducted a randomized, double-blind, phase II study, investigating vandetanib (100 mg/day) *vs.* placebo, in combination with docetaxel (75 mg/m² every three weeks) and prednisolone (10 mg/day). This study included 86 patients with metastatic HRPC. The primary endpoint was PSA response (>50% reduction in plasma levels). The results showed an increase in adverse events (disease progression or death) in the vandetanib arm (65% *vs*. 60%, HR = 1.13, p = 0.67), though this was not statistically significant. The overall incidence of adverse events was similar in both groups. The safety and tolerability profile for vandetanib was similar to that previously reported. In this study, vandetanib associated with docetaxel/prenisolone gave no increase in efficacy based on the PSA level, *vs.* placebo combined with docetaxel/prednisolone.

## 6. Synopsis and Conclusion

EGFR targeting in HRPC is a logical approach as HER1 is very frequently overexpressed in this disease, and activation of EGFR leads to cell proliferation, neoangiogenesis, and an increasedtumor invasion due tothe greater mobility of these tumor cells. Moreover, pharmaceutical agents in this category are now well known and most have been shown to induce clinical benefits in the treatment of solid tumors. Although preclinical findings on the use of anti-EGFR molecules (either monoclonal antibodies or TKI) were promising, the transition to clinical phases as monotherapy has been rather disappointing. However, clinical approaches in which anti-EGFRs are combined with antimitotics (and in particular with docetaxel) must be continued. This use of combined treatments with targeted therapies such as those targeting EGFR may improve dose adjustment of the systemic cytotoxic drugs administered in a usually elderly population with multiple comorbid diseases and an aggressive cancerous disease. The preclinical results also suggest that combination of other targeted therapies with EGFR and in particular antiangiogenics should be considered. Multi-target TKI may also be useful. However, the main limitation will be the toxicity of this multi-agent therapy, all the more as HRPC often affects elderly and vulnerable populations. Prostate cancer is therefore a good model for geriatric oncology approaches. Clinical studies must therefore integrate geriatric evaluation parameters, in order to better define those patients for whom treatment will be really beneficial. These parameters concern cognitive status, nutritional status, signs of anxiety/depression, the evaluation of the number and severity of comorbidities, drug misadventures related to polymedication, autonomy during daily activities, the risk of falls and socio-environmental factors determining the quality of patient support and care. In this setting, survival should perhaps not be the main parameter used to assess studies, although this requires a cultural change extolling a geriatric oncology approach. This approach is now recommended by the International Society of Geriatric Oncology (SIOG) which recommends that therapeutic strategies are decided after a geriatric assessment for vulnerable patients [53].
